# A chromosome-level genome assembly for the astaxanthin-producing microalga *Haematococcus pluvialis*

**DOI:** 10.1038/s41597-023-02427-1

**Published:** 2023-08-03

**Authors:** Chao Bian, Chenglong Liu, Guiying Zhang, Ming Tao, Danqiong Huang, Chaogang Wang, Sulin Lou, Hui Li, Qiong Shi, Zhangli Hu

**Affiliations:** 1 Shenzhen Engineering Laboratory for Marine Algal Biotechnology, Guangdong Technology Research Center for Marine Algal Bioengineering, Longhua Innovation Institute for Biotechnology, College of Life Sciences and Oceanography, Shenzhen University, Shenzhen, 518055 China; 2 Key Laboratory of Optoelectronic Devices and Systems of Ministry of Education and Guangdong Province, College of Optoelectronic Engineering, Shenzhen University, Shenzhen, 518060 China; 3 Shenzhen Key Lab of Marine Genomics, BGI Academy of Marine Sciences, BGI marine, Shenzhen, 518081 China; 4 School of Archaeology and Museology, Sichuan University, Chengdu, 610064 China; 5 Center for Archaeological Science, Sichuan University, Chengdu, 610064 China

**Keywords:** Genome, Plant genetics

## Abstract

The green microalga *Haematococcus pluvialis* can synthesize high amounts of astaxanthin, which is a valuable antioxidant that has been utilized in human health, cosmetics, and aquaculture. To illustrate detailed molecular clues to astaxanthin yield, we performed PacBio HIFI along with Hi-C sequencing to construct an improved chromosome-level haplotypic genome assembly with 32 chromosomes and a genome size of 316.0 Mb. Its scaffold N50 (942.6 kb) and contig N50 (304.8 kb) have been upgraded remarkably from our previous genome draft, and a total of 32,416 protein-coding genes were predicted. We also established a high-evidence phylogenetic tree from seven representative algae species, with the main aim to calculate their divergence times and identify expanded/contracted gene families. We also characterized genome-wide localizations on chromosomes of some important genes such as five *BKTs* (encoding beta-carotene ketolases) that are putatively involved in astaxanthin production. In summary, we reported the first chromosome-scale map of *H. pluvialis*, which provides a valuable genetic resource for in-depth biomedical investigations on this momentous green alga and commercial astaxanthin bioproduction.

## Background & Summary

**Fig. 1:**
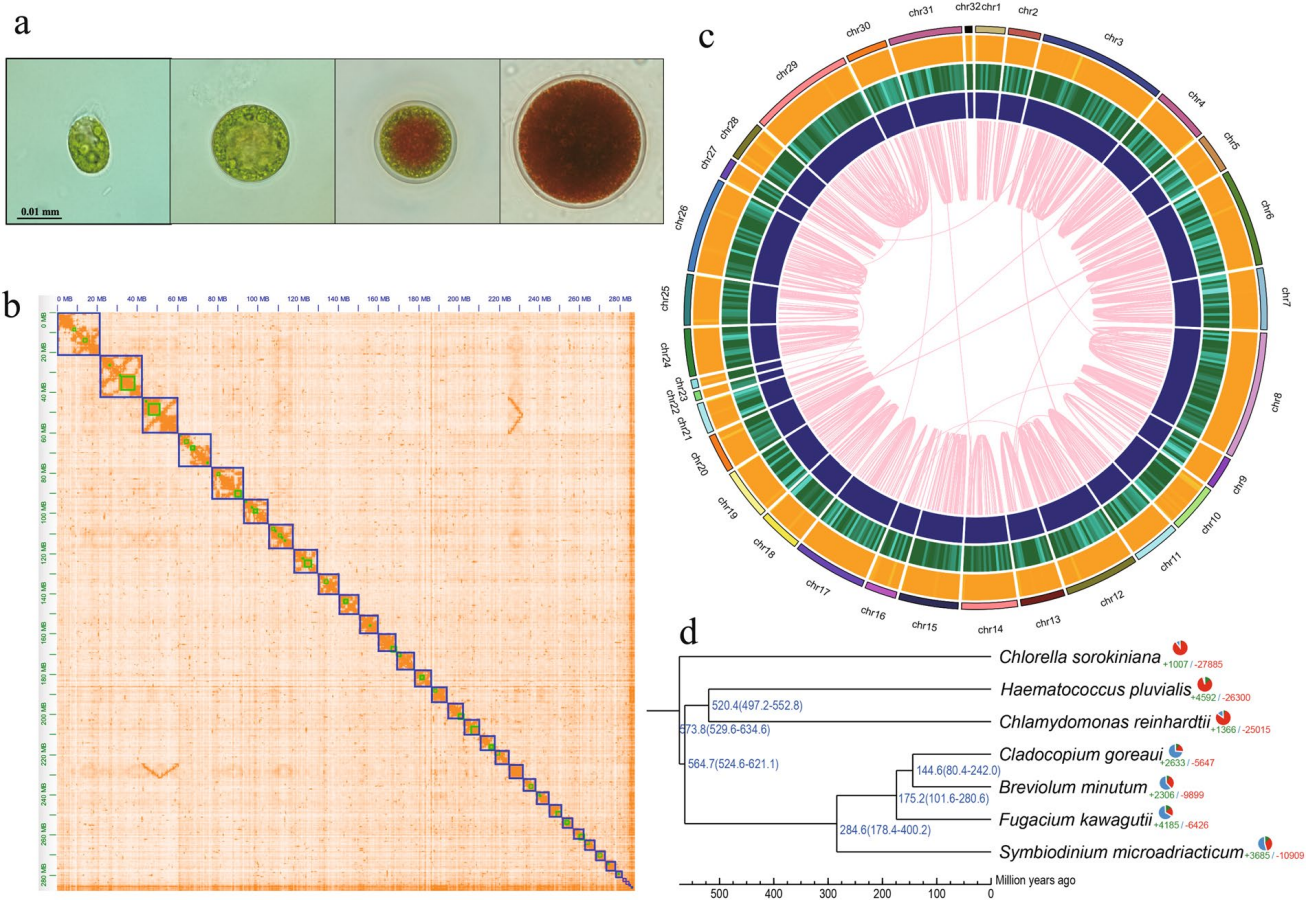
*H. pluvialis* and its genome. (**a**) Images of the *H. pluvialis* life cycle, inclduing macrozooid, palmella, immature aplanospore and aplanospore (from left to right), respectively. (**b**) The heatmap view of Hi-C result. (**c**) The circos view of *H. pluvialis* genome. (**d**) Phylogeny and gene-family analysis of seven representative microalgae species.

The freshwater unicellular green microalga *Haematococcus pluvialis* is well known as the best natural bioresource for production of the carotenoid astaxanthin. It has attracted a lot of attention frequently due to its high capacity to synthesize astaxanthin, which is of high value with strong pharmaceutical activity for commercial industries^1^. The intriguing life cycle of *H. pluvialis* includes four stages of distinguishable cellular morphologies, i.e., macrozooid, palmella, immature aplanospore and aplanospore (from left to right in Fig. 1a). It maintains the green motile stage at favorable environmental conditions. However, when it experiences unfavorable environmental or stress conditions, the cells of *H. pluvialis* change into red immobile cells (also named as cysts). Meanwhile, these cells also expand cell size, lose flagella, produce astaxanthin, and build thick cell walls^2^. On the other hand, during their vegetative growth, *H. pluvialis* cells are spherical, ellipsoidal, and pear-shaped with flagella and chloroplasts (see more details in the images of Fig. 1a).

Previous studies with transcriptomics, metabolomics and/or proteomics data have identified several important genes related to astaxanthin biosynthesis under stress conditions (such as high irradiation, nitrogen deprivation, and nutrient starvation)^3–5^. In our previous report^6^, a draft genome assembly was generated with assistance of only Illumina short-read sequencing. However, its contigs and scaffolds are fragmental, resulting in somewhat redundancy. That assembly is 669.0 Mb in length, with relatively low values of scaffold N50 (288.6 kb) and contig N50 (8.4 kb)^6^. Due to fragmental assembly and limited genome resources, details of molecular clues to astaxanthin biosynthesis in *H. pluvialis* remain elusive. Here, we performed long-read PacBio HIFI and high-resolution chromosome conformation capture (Hi-C) sequencing, with the main aim to obtain a high-quality and chromosome-level genome assembly of *H. pluvialis*.

Whole-genome sequencing, assembly, and annotation of this economically important microalga were fulfilled with a great improvement. In addition, carotene biosynthetic genes cooperate with β-carotene ketolase (CRTO) and hydroxylase (CRTR-B) to synthesize astaxanthin under high irradiation and salinity stress, which are the most common stressors during cultivation of *H. pluvialis*^3–5^. We therefore conducted additional transcriptome analysis on stressed cells to reveal differential astaxanthin production from certain critical genes, such as those encoding beta-carotene ketolases (*BKT*s)^6^. In the coming future, these valuable genomic resources will facilitate breeding of novel *H. pluvialis* strains to obtain higher astaxanthin yield.

## Methods

### Sample materials, whole genome sequencing and genome assembly

The *H. pluvialis* strain 192.80 was purchased from the SAG Culture Collection of Algae (Göttingen University, Göttingen, Germany). Microalga cells were cultivated in ESP Ag medium^7^. Genomic DNAs were extracted from cultivated cells using Qiagen GenomicTip100 (Qiagen, Germantown, MD, USA). A routine whole-genome shotgun sequencing strategy was applied. In brief, long SMRTbell libraries were constructed for the HiFi sequencing based on PacBio’s standard protocol (Pacific Biosciences, Menlo Park, CA, USA). These libraries were sequenced through a PacBio Sequel II System (Pacific Biosciences). About 1.6 million of consensus reads (26.1 Gb) with a mean length of 16.6 kb were generated. For the Hi-C sequencing, genomic DNAs were fixed with formaldehyde, sheared by a restriction enzyme (MboI) to build a Hi-C library, and then sequenced on a HiSeq-Xten sequencing platform (Illumina Inc., San Diego, CA, USA). A total of 98.0 Gb of 150-bp paired-end Hi-C data were generated.

**Fig. 2:**
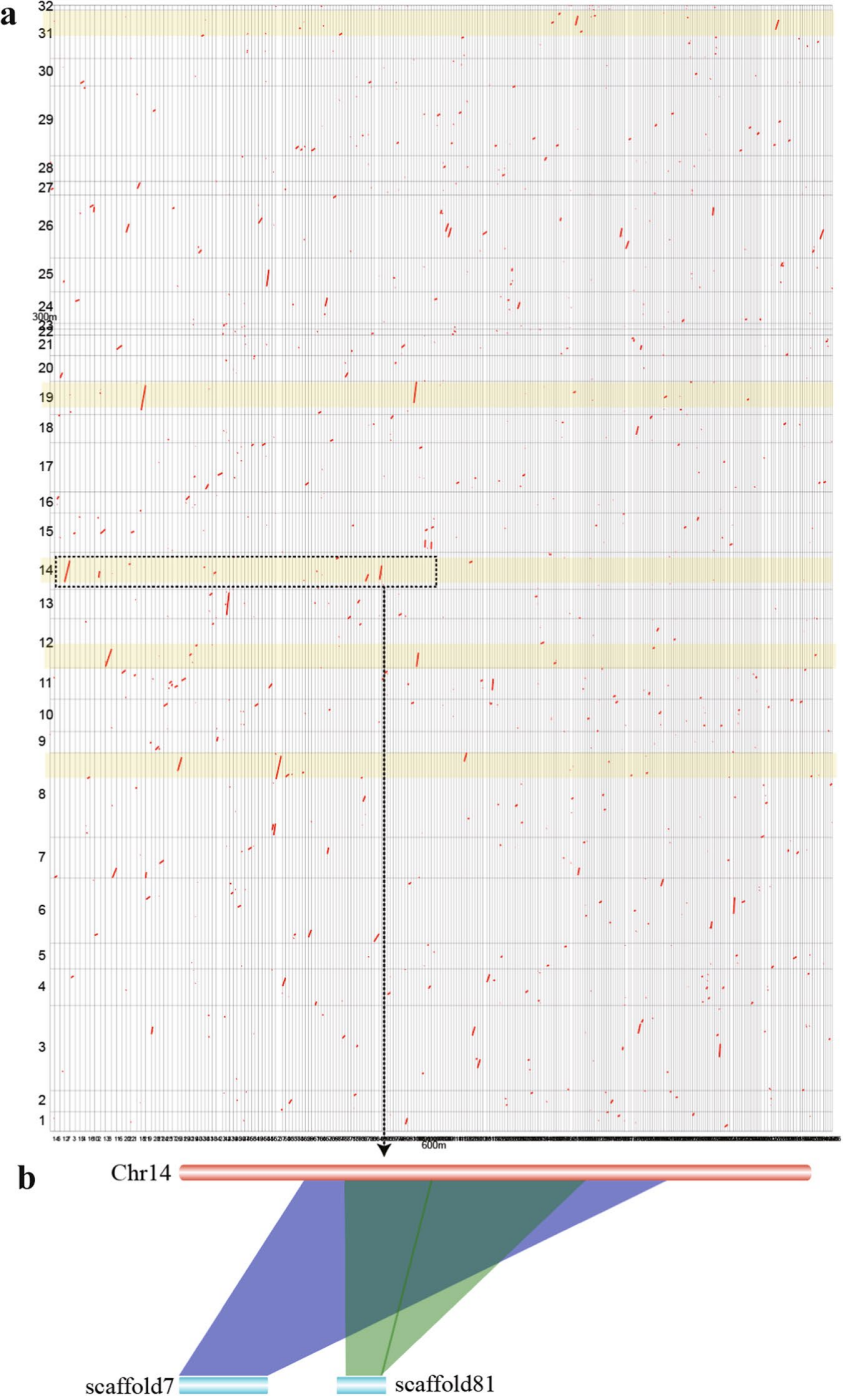
Synteny blocks between our current chromosome-level assembly and the previous 600-Mb draft^6^ of the *H. pluvialis* genome. (**a**) An overview of the total synteny blocks. Only partial blocks are visible due to the fragmental contigs in the previous draft assembly. (**b**) An example of the solid 1:2 blocks in Chr14.

The sequenced HIFI reads were initially assembled to be contigs by hifiasm (version 0.14-r312)^8^. This primary genome assembly contains 315.1 Mb of contigs, with a N50 length of 304.8 kb (Table 1). Subsequently, we integrated Hi-C data to construct a high-quality *de novo* assembly at the chromosome level. In brief, quality control was performed to filter the Hi-C raw reads, and then valid Hi-C connected reads were generated by Juicer version 1.5^9^. The 3D *de novo* assembly (3D-DNA, version 180922) pipeline^10^ was employed to link the contig sequences into chromosome-level sequences. Haplotypic 32 chromosomes (chr; Fig. 1b) with a total length of 285.4 Mb (Table 2) were built by using these Hi-C sequences; they account for about 90.3% of the whole genome assembly (Fig. 1c), individually ranging from 1.2 Mb (Chr32) to 21.5 Mb (Chr8) in length (Table 2). The completeness of this final genome assembly was evaluated by Benchmarking Universal Single-Copy Orthologs (BUSCO)^11^ v5.2.2, revealing that 93.4% BUSCOs are complete. We also compared this assembly (~300 Mb) with our previous draft assembly^6^ (~600 Mb), and found a lot of 1:2 blocks (Fig. 2a) by using the i-ADHoRe v3.0 software^12^. For example, two scaffolds (7 and 81) in the draft genome correspond to the Chr14 in part (see Fig. 2b).

**Table 1:** Summary of the primary genome assembly and annotation.

Genome assembly	Data
Contig N50 size (kb)	304.8
Scaffold N50 size (kb)	942.6
Assembled genome size (kb)	315,985.9
Genome coverage (×)	82.9
Longest scaffold (kb)	21,607.0
**Genome annotation**	
Number of protein-coding genes	30,575
Transposable elements content (%)	50.8

**Table 2:** Chromosome length and astaxanthin biosynthesis related genes in the assembled *H. pluvialis* genome.

Chr ID	Length (bp)	Related genes^†^	Chr ID	Length (bp)	Related genes^†^
Chr1	4,970,000		Chr17	12,486,648	
Chr2	5,430,000		Chr18	7,151,099	
Chr3	2,1607,000		Chr19	8,564,122	*LCYB* (4)
Chr4	9,297,664		Chr20	6,515,030	
Chr5	6,569,691		Chr21	5,182,000	
Chr6	16,549,500		Chr22	1,515,144	
Chr7	10,396,840		Chr23	1,472,754	
Chr8	21,499,393	*ZDS1* (2)	Chr24	8,050,159	
Chr9	5,457,637		Chr25	8,590,500	*CHYB* (3)
Chr10	8,246,294	*BKT* (1)	Chr26	16,037,447	*BKT* (3)
Chr11	7,935,624		Chr27	3,462,500	
Chr12	12,546,000		Chr28	6,605,496	
Chr13	7,467,357	*CHYB* (4)	Chr29	17,722,355	*PSY* (2)
Chr14	9,426,245		Chr30	6,974,760	*PSY* (1)
Chr15	10,026,745		Chr31	12,357,645	
Chr16	5,331,004		Chr32	1,189,999	

^†^These putative astaxanthin biosynthesis related genes encode beta-carotene ketolase (*BKT*), Beta-carotenoid hydroxylase (*CHYB*), Lycopene beta-cyclase (*LCYB*), phytoene synthase (*PSY*) and Zeta-carotene desaturase 1 (*ZDS1*), respectively. Gene numbers are provided in the brackets.

### Genome annotation

Repeat sequences of the assembled *H. pluvialis* genome were identified by employing several programs including Tandem Repeats Finder^13^, LTR_FINDER^14^, RepeatProteinMask and RepeatMasker^15^. Tandem Repeats Finder^13^ was employed to search for tandem repeats using optimized parameters (Match = 2, Mismatch = 7, Delta = 7, PM = 80, PI = 10, Minscore = 50, and MaxPerid = 2,000). A *de novo* repeat library was built by the LTR_FINDER (version 1.0.6; parameter: -w 2). RepeatMasker was then utilized to map the *H. pluvialis* assembly onto the Repbase TE library (version 3.2.9)^16^ so as to search for known repeat sequences as well as map it onto the *de novo* repeat libraries to identify novel types of repeat sequences.

We then performed annotation of the *H. pluvialis* genome assembly with two routine methods, including homology-based and transcriptome-based annotation. Eight representative species, including *Chlamydomonas reinhardtii*, *Paramecium tetraurelia*, *Saccharomyces cerevisiae*, *Symbiodinium kawagutii*, *Symbiodinium minutum*, *Chlamydomonas eustigma*, *Chromochloris zofingiensis* and *Micromonas pusilla*, were downloaded from NCBI and were selected for the homology annotation. Their protein sequences were mapped onto the *H. pluvialis* genome assembly utilizing TblastN^17^ with an E-value ≤ 1e^−5^. Genewise 2.2.0^18^ was subsequently employed to predict gene structures based on these TblastN results. Total RNA was extracted from those control cells (sample ID: LLMT4, 5, and 6; see more details in the followed section on Total RNA Isolation) for subsequent transcriptome sequencing on an Illumina HiSeq4000 platform. These data were mapped onto the assembled genome using HISAT v2.0.4^19^. We then utilized Cufflinks (version 2.2.1)^20^ to identify those preliminary genes. Finally, we applied Maker^21^ to integrate predicted genes from both annotation procedures.

The final gene set is composed of 32,416 protein-coding genes, with an average of 6.3 kb in length. Their deduced protein sequences were then mapped against public TrEMBL, Swiss-Prot^22^, InterProScan^23,24^, KEGG^25^ and NR databases using BlastP with an E-value ≤ 1e^−5^. Finally, approximately 97.2% of the predicted genes have at least one functional assignment from these public databases.

### Transcriptome analysis

The *H. pluvialis* strain 192.80 was purchased and cultured by using Bold Basal Medium in 250-mL Erlenmeyer flasks at 22 °C under continuous fluorescent lamps (20 μmol·m^−2^·s^−1^) to the logarithmic phase (about 1 × 10^5^ cells·mL^−1^)^6^. Algal cells were sub-cultured into 300 mL BBM medium and treated with salicylic acid (SA, 25 mg·L^−1^) and high light (HL, 350 μmol·m^−2^·s^−1^). The treatment of salicylic acid and high light was named as SAHL for short. Algal cells after treatments with at 0 h (Control), 1 h (SAHL 1), 6 h (SAHL 6), 12 h (SAHL 12), 24 h (SAHL 24), and 48 h (SAHL 48) were collected and used for RNA-seq sampling, respectively. These transcriptome reads were dowanloaded from previous study^26^ with the accession number PRJNA675306. Paired-end raw reads were then processed by removal of adapters and low-quality sequences using SOAPnuke (version 1.5.6)^27^ with default parameters. These clean data were then mapped onto the assembled genome using HISAT^19^.

Transcript quantification of fragments per kilobase per million (FPKM) in each sample was calculated by using Cufflink^28^. Differentially expressed genes (DEGs) between the treatment and control groups were identified using “edgeR” (version 3.15) in the R package^29^ with log2 (ratio) ≥ 1 and adjusted P-value ≤ 0.05 as the threshold. Finally, a pathway enrichment analysis was conducted on these up- and down-regulated DEGs according to the KEGG database^25^.

### Evolutionary placement of *H. pluvialis*

A whole-genome phylogenetic analysis on *H. pluvialis* and other six related microalgae was performed to determine the evolutionary position of *H. pluvialis*. These examined species, including *Chlorella sorokiniana*, *Chlamydomonas reinhardtii*, *Cladocopium goreaui*, *Breviolum minutum*, *Fugacium kawagutii* and *Symbiodinium microadriacticum*, were downloaded from NCBI. The whole-genome gene sets from *H. pluvialis* and others were aligned by BLASTp (version 2.2.6) to check homology for generation of a sequence similarity matrix, which was then utilized to identify gene families by using OrthoMCL^30^ and Markov Chain Clustering (MCL) with default parameters. We identified single-copy orthologues among the seven species, and then these orthologues were aligned with MUSCLE version 3.7^31^. All alignments were combined to obtain a super alignment sequence.

We first applied the maximum likelihood (ML) method to generate a phylogenetic tree (Fig. 1d), which was implemented in PhyML version 3.0^32^. To confirm this topology, we also utilized Bayesian inference (BI) to establish the same phylogenetic tree by using MrBayes version 3.2.2^33^. Meanwhile, we calculated the divergence time by MCMCtree in the PAML package^34^, with calibration references from the TIMETREE^35^. Expanded and contracted gene families were predicted by employing CAFE v4.2.1^36^. In total, 41 single-copy gene families including 287 genes were identified from the seven representative microalgal species. These genes from each species were concatenated together and finally constituted a super-length nucleotide dataset to yield 165,027 aligned sites. Our phylogenetic analysis and divergence results discovered that *H. pluvialis* is close to *C*. *reinhardtii*, and their divergence time is at 520.4 million years ago (Mya). We found 4,592 expanded gene families and 26,300 contracted gene families in the *H. pluvialis* genome (Fig. 1d).

### Localization of some astaxanthin biosynthesis related genes in the *H. pluvialis* genome

Protein sequences of BKT (accession no. CAA60478.1, Beta-carotene ketolase), PSY (CHLRE_02g095092v5, Phytoene synthase), PDS (CHLRE_12g509650v5, Phytoene desaturase), ZDS1 (CHLRE_07g314150v5, Zeta-carotene desaturase 1), LCYB (CHLRE_08g358538v5, Lycopene beta-cyclase), and CHYB (CHLRE_04g215050v5, Beta-carotenoid hydroxylase) were downloaded from the NCBI. We utilized tBLASTn (version 2.2.6) to search the coding regions of these putative astaxanthin biosynthesis related genes^6^, and their encoding sequences in the *H. pluvialis* genome were further predicted by Genewise2.2.0^18^.

*H. pluvialis* is popular for its strong capacity to produce large amounts of astaxanthin that is a strong antioxidant for human health, cosmetics and aquaculture^1^.

**Fig. 3:**
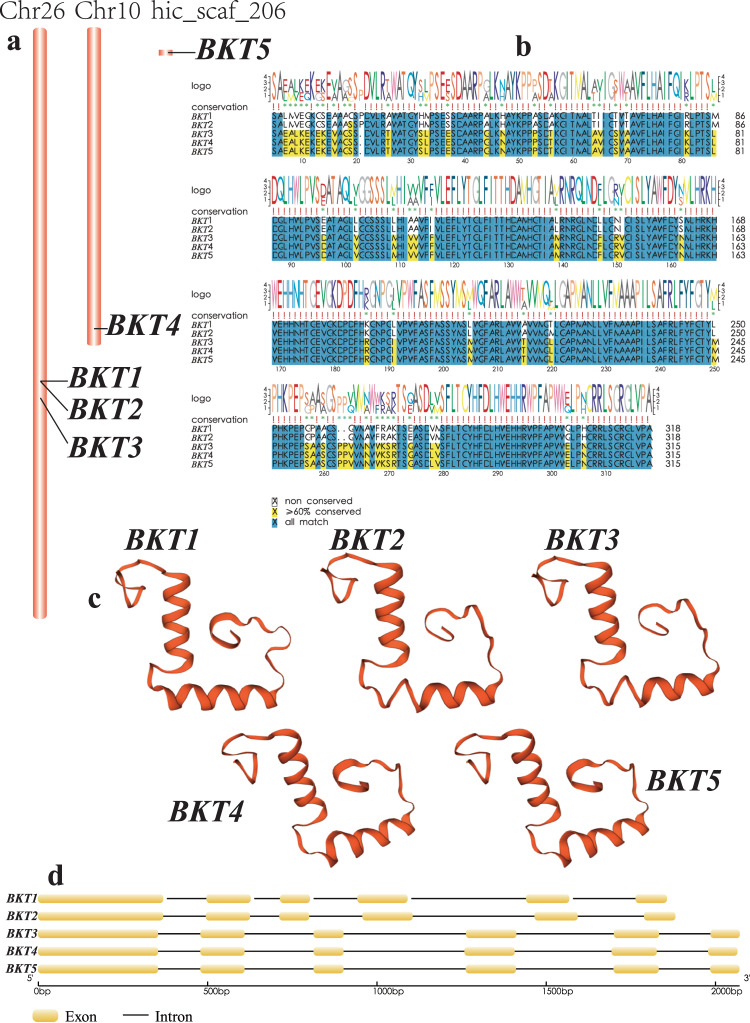
Summary of the five *bkt* genes in *Haematococcus pluvialis*. (**a**) The chromosome location of *BKT*1-5. (**b**) Alignment of the five BKT protein sequences. (**c**) 3D structures of the five BKT proteins. (**d**) Distribution of exons and introns.

Because about 1 kg of dry *H. pluvialis* cells can produce over 40 g astaxanthin^37^, this species is a great material for production of astaxanthin. Previous study has shown that CRTO and CRTR-b are two key enzymes for the astaxanthin biosynthesis pathway^38^. In this study, three BKT genes were identified to be up-regulated in *H. pluvialis* cells with diverse stress treatments^39^. However, in our current genome searching, we identified five *BKT* genes that were distributed in Chr10, Chr26 and scaffold206 (Fig. 3a). More excitingly, a tandem duplication of three *BKT* genes (*BKT1*, *BKT2* and *BKT3*) were observed in the Chr26. These *BKT* genes have similar protein sequences (Figs. 3b), 3D structures (Fig. 3c) and gene structures (Fig. 3d), while with potentially different astaxanthin-producing capacity. For example, we qualified *BKT3* with the highest transcription level (data not shown) under the SAHLtreatment (see the transcriptome sequencing section and the previous report^26^). We therefore propose that some of these duplicated *BKT* genes (*BKT1*, *2* and *3*) are potetntially the major contributors to the rapid synthesis and accumulation of large amounts of astaxanthin. Conversely, the relative *C. reinhardtii*, without evidence of astaxanthin production, doesn’t have any functional *BKT* gene in its genome.

On the other hand, we compared several other genes from the astaxanthin biosynthesis pathway, and observed that some in *H. pluvialis* are remarkably expanded than *C. reinhardtii*. For example, the *H. pluvialis* genome contains 8 *CHYB*, 3 *PSY*, 3 *ZDS1*, 2 *LCYB* and 1 *PDS* genes, corresponding to only one of each in the *C. reinhardtii* genome; these high copy numbers may provide additional support for the high astaxanthin production in *H. pluvialis*.

In summary, we reported the first chromosome-level whole-genome assembly for the attractive astaxanthin-producing green microalga *H. pluvialis*. This genome is a valuable material for deep understanding the molecular clue of algal astaxanthin yield. We found five *BKT* genes in *H. pluvialis* genome. These expanded genes may play a key role in the high astaxanthin production. Our genome and transcriptome data sets will facilitate molecular breeding or biosynthesis of novel strains with significantly improved astaxanthin contents.

## Data Records

The PacBio long reads, Hi-C sequencing reads, and the final genome assembly were deposited at NCBI with the accession number PRJNA964479^40^. The annotation data and protein sequences were deposited at Figshare with doi number 10.6084/m9.figshare.23047088^41^. The raw reads were deposited at NCBI Sequence Read Archive with accession number SRR25425436 and SRR25425436^42^.

## Technical Validation

The extracted DNA quality was examined by using the agarose gel electrophoresis with over 1.8 of the DNA spectrophotometer ratios (260/280) and around 20 kb main band. The Nanodrop ND-1000 spectrophotometer (RIN > 8.0; LabTech, Corinth, MS, USA) was utilized to check the purified RNA quality. The completeness of this *H. pluvialis* genome was validated by BUSCO v5.2.2. The final BUSCO result showed 93.4% completeness.

## Data Availability

The execution of all software and pipelines in this study strictly followed the manuals and protocols of the published bioinformatic tools. The versions of the software employed have been specified in the Methods section. If no parameter is provided, the default is used. No custom code was employed.
